# Adult Onset Still's Disease and Autoinflammation

**DOI:** 10.1155/2012/964751

**Published:** 2012-08-29

**Authors:** Petros Efthimiou, L. Nandini Moorthy, Clio P. Mavragani, Dimitris Skokos, Bruno Fautrel

**Affiliations:** ^1^Rheumatology Division, Lincoln Medical & Mental Health Center, 234 E. 149th st, New York, NY 10451, USA; ^2^ Department of Medicine, Weill Cornell Medical College, New York, NY 10021, USA; ^3^Division of Pediatric Rheumatology, Robert Wood Johnson Medical School, University of Medicine and Dentistry of New Jersey, 1 Robert Wood Johnson Place, New Brunswick, NJ 08901, USA; ^4^Department of Physiology, School of Medicine, University of Athens, M. Asias 75, 11527 Athens, Greece; ^5^Immunity & Inflammation Department, Regeneron Pharmaceuticals, Inc, 777 Old Saw Mill River Road Tarrytown, NY 10591, USA; ^6^Pierre et Marie Curie University (Paris 6), Service de Rhumatologie, Groupe Hospitalier Pitié-Salpêtrière, 83 Boulevard de l'Hôpital, 75013 Paris, France

## 1. Introduction

The goal of this special issue is to present, in a comprehensive fashion, the latest data on Adult onset Still's Disease, within the broader context of the current concepts of autoinflammatory diseases and the immune mechanisms associated with them. A detailed review of Th-17 immune mechanisms and their association with autoinflammation by Waite and Skokos [1] is followed by two articles on potential disease biomarkers, serum ferritin, and IL-18 by Mehta and Efthimiou [2] and Colafrancesco et al., respectively. Mavragani et al. contributed with an up-to-date comprehensive report of Adult Still's, while Gurion et al. [3] go in depth over its pediatric counterpart, systemic JIA. Rossi-Semerano et al. examine whether both entities fall within the autoinflammatory spectrum and Giampietro et al. provide us with a detailed account of the leading treatments targeting IL-1, the common denominator.

## 2. Adult-Onset Still's Disease

Adult-onset Still's disease (AOSD) is a rare autoinflammatory disorder of unknown etiology, which was initially described in adults by Eric Bywaters in 1971, who also coined the term (AOSD) due to the disease's close resemblance to a pediatric syndrome described by Dr. George Still in 1899, currently known as systemic juvenile idiopathic arthritis (sJIA) [4]. During the first forty years, the pathophysiology of the disease had remained largely obscure and only recently was our understanding of the disease enhanced by the description of the autoinflammatory syndromes. The term “autoinflammatory” has been ascribed to a group of disorders characterized by frequent attacks of inflammation without any indication that this process is related to auto-antigen stimulus. These disorders are associated with defective interleukin-1 processing, regulation of nuclear factor-B transcription factor, and likely abnormal cellular apoptosis. Mutations in genes encoding the tumor necrosis factor (TNF) receptor and pyrin superfamilies of molecules may result in the endurance of leukocytes that would customarily go through apoptosis. As a result, relatively minor proinflammatory triggers may lead to an exaggerated, and potentially harmful, inflammatory response. Patients with autoinflammatory syndromes, including the classic hereditary periodic fever syndromes, may share certain genetic traits; the MEFV gene mutation M694V, associated with familial mediterranean fever (FMF) and IL-1 hypersecretion was seen with increased frequency in turkish children with sJIA. Furthermore, macrophage activating syndrome (MAS), a severe, life-threatening complication that is particularly frequent in patients with AOSD and sJIA has been associated with mutations in the perforin [5] and the MUNC13-4 genes [6].

The diagnosis of AOSD continues to be a clinical one and, in the absence of a definitive diagnostic test, often necessititates the arduous exclusion of potential mimickers, that is, infectious, neoplasmatic, autoimmune, and other autoinflammatory diseases and can be facilitated by the use of one of several validated diagnostic criteria, that is Yamaguchi's, Cush's, or Fautrel's [7–9].

Disease severity varies significantly among affected individuals and, even, within the same individual. In certain cases, a single or infrequent recurrent flares, usually dominated by systemic symptoms (fever, rash) that can mask the concurrent inflammatory polyarthritis, may resolve spontaneously or require a short course of systemic corticosteroids [10]. On the more severe end of the spectrum, multiple flares of debilitating frequency or continously active disease, often associated with chronic progressive arthritis and disability, require continous, aggressive immunomodulatory treatment and are associated with complications that carry significant morbidity and mortality.

 Systemic corticosteroid therapy is still touted as the primary treatment, especially targeting systemic manifestations, despite concerns regarding the risks associated with their long-term use and their efficacy in preventing radiographic progression of chronic inflammatory arthritis. Tradiitional disease modifying antirheumatic drugs (DMARDs), especially methotrexate (MTX), have shown efficacy in inducing remission in refractory cases and are frequently used as steroid sparing drugs and, also, for prevention of arthritis progression, ankylosis, and disability. However, many cases prove to be refractory and their management remains a challenge; clinicians find themselves combating a disease with protean manifestations with limited evidence-based guidance caused by a paucity of controlled studies [11]. Moreover, there is a growing number of published reports describing rare, albeit life-threatening, multisystemic complications of AOSD [5, 6, 12–14]. Elevated levels of proinflammatory cytokines such as IL-1*β*, IL-6, IL-17, IL-8, IL-18, and TNF-a were previously described in AOSD patients, often in association with disease activity and/or distinct clinical phenotypes and serological feaures such as hepatic involvement, arthritic complaints, salmon rash, and hyperferittinimia among others [15–21]. Given these findings, available biologic agents targeting IL-1, IL-6, and TNF-a for other rheumatologic conditions led to their off-label use in AOSD, with variable success.

 While antiTNF agents have been proved moderately efficacious in AOSD refractory cases particularly in the chronic articular form of the disease [22, 23], IL-1 inhibition is currently considered the mainstay of treatment for AOSD leading to significant improvement in both clinical and laboratory terms [15, 24]. Preliminary results from case series also support a role for IL-6 blockade in the management of refractory disease forms, given the implication of this cytokine in disease pathogenesis [25–30]. Finally, and since the contributory role of T-cell compartment has been increasingly recognized in AOSD pathophysiology, abatacept—a T-cell costimulation stimulator—has been succesfully used in refractory AOSD cases [31, 32]. Identification of distinct pathogenetic pathways and association with clinical and serological phenotypes would allow the design of rational, tailored therapies for AOSD management.

## 3. Pediatric Still's Disease or Systemic Onset Juvenile Idiopathic Arthritis?

Still's disease in children comprising fevers, arthritis, rash, widespread adenopathy, and serositis, splenomegaly, and elevation of acute phase reactants has been termed systemic juvenile rheumatoid arthritis, systemic juvenile chronic arthritis, and is now called systemic arthritis according to the International League of Associations of Rheumatology (ILAR) classification for juvenile idiopathic arthritis (JIA) [1, 2].

This is an unfortunate choice as the scientific evidence suggests that systemic onset disease has no relationship to the other forms of juvenile idiopathic arthritis. sJIA has a distinctly different epidemiology, natural history, cytokine profile, and pathogenesis. Thus inclusion of children with sJIA in studies of every type results in increased probability of erroneous conclusions. Innate immune abnormalities in sJIA suggest it is more appropriately considered among the autoinflammatory diseases [3]. At the fourth international congress on the systemic autoinflammatory diseases, sJIA was described as a complex or multifactorial autoinflammatory disease [4].

Furthermore, in contrast to polyarticular JIA and juvenile ankylosing spondylitis, sJIA and autoinflammatory diseases are not associated with major histocompatibility complexes [3]. Unlike polyarticular, oligoarticular or psoriatic JIA, where there is a female predominance, and juvenile ankylosing spondyliltis occurring more commonly in males, in sJIA, there is no distinct gender predilection.

The differences in pathogenesis and clinical features argue that sJIA should be considered primarily as an autoinflammatory syndrome. In the current nomenclature system, we should recognize sJIA as being distinct from other subtypes of JIA in terms of its pathogenesis, genetics, gender predilection, and treatment. As a result children with sJIA are not appropriately included in studies of the natural history, pathogenesis or treatment of JIA, but must be considered separately if we are to truly improve our understanding.

## 4. Research Agenda

Despite the recent advancements in cytokine biology, our understanding of the pathogenesis of AOSD, especially the inflammatory pathways and the influence of environmental factors at the origin of the autoinflammatory cascade is still at its infancy. We have not yet identified the responsible environmental factors that may trigger autoinflammation and neither have we any indicator of who may be more susceptible to these external factors. Are those environmental factors the same for the pediatric and adult disease forms, and if so, what causes the clinical manifestations to appear with higher frequency in children (sJIA) or young adults (AOSD) compared to older individuals? In this direction, further studies are needed to elucidate the potential relationship between AOSD and sJIA and the, better characterized, hereditary autoinflammatory syndromes (e.g., TRAPS or hyper-IgD syndrome). Finally, controlled trials would help to define optimal strategies, especially for conventional treatment or biologic agents, in order to reduce the use of, often prolonged, high-dose corticosteroid therapy that is associated with severe side effects. In order for these studies to become reality, the formation of national and international research networks is an absolute necessity due to the disease characteristics: the disease is rare, the clinical presentation is heterogeneous, and patients are often cared for by different specialists. Uniform procedures could increase experience sharing and enable better knowledge integration.
